# 3D Ultrastructural Organization of Whole *Chlamydomonas reinhardtii* Cells Studied by Nanoscale Soft X-Ray Tomography

**DOI:** 10.1371/journal.pone.0053293

**Published:** 2012-12-31

**Authors:** Eric Hummel, Peter Guttmann, Stephan Werner, Basel Tarek, Gerd Schneider, Michael Kunz, Achilleas S. Frangakis, Benedikt Westermann

**Affiliations:** 1 Institut für Zellbiologie, Universität Bayreuth, Bayreuth, Germany; 2 Helmholtz-Zentrum für Materialien und Energie GmbH, Institute for Soft Matter and Functional Materials, Berlin, Germany; 3 Frankfurt Institute for Molecular Life Sciences and Institute of Biophysics, Goethe University Frankfurt, Frankfurt am Main, Germany; University of Hyderabad, India

## Abstract

The complex architecture of their structural elements and compartments is a hallmark of eukaryotic cells. The creation of high resolution models of whole cells has been limited by the relatively low resolution of conventional light microscopes and the requirement for ultrathin sections in transmission electron microscopy. We used soft x-ray tomography to study the 3D ultrastructural organization of whole cells of the unicellular green alga *Chlamydomonas reinhardtii* at unprecedented spatial resolution. Intact frozen hydrated cells were imaged using the natural x-ray absorption contrast of the sample without any staining. We applied different fiducial-based and fiducial-less alignment procedures for the 3D reconstructions. The reconstructed 3D volumes of the cells show features down to 30 nm in size. The whole cell tomograms reveal ultrastructural details such as nuclear envelope membranes, thylakoids, basal apparatus, and flagellar microtubule doublets. In addition, the x-ray tomograms provide quantitative data from the cell architecture. Therefore, nanoscale soft x-ray tomography is a new valuable tool for numerous qualitative and quantitative applications in plant cell biology.

## Introduction

The availability of three-dimensional high resolution microscopy is vital for life science. While the resolution of conventional light microscopes is limited by the wavelength of the light, super resolution light microscopy, such as STED and Palm, is able to push the resolution limit down to the nanoscale range [1]. However, its use is limited to the visualization of fluorescently labeled structures, rather than whole cells. On the other hand, electron tomography is increasingly used to analyze the ultrastructural organization of cells with a resolution in the nanometer range [2]–[4]. However, only bacteria or exceptionally small eukaryotic cells can be analyzed in a single electron tomogram [5]. For the vast majority of eukaryotic cell types serial sectioning and acquisition of several tilt-series is needed to cover the entire cell. Soft x-ray microscopy is a new technique that closes the gap between light and electron microscopy currently providing a spatial resolution of 11 nm (half-pitch) [6]. Importantly, whole cells with a thickness of up to 15 µm can be imaged in single tomograms, thus eliminating the limitations of transmission electron tomography. On a whole cell approach the HZB-TXM at the electron storage ring BESSY II is able to achieve a 3D resolution of 36 nm [7].

Soft x-ray microscopy provides a number of unique advantages: First, frozen hydrated samples used for x-ray microscopy are in a close to native state. They can be imaged without the need for staining with heavy metals or fluorescent markers. Image contrast in the water window wavelength range between the K-shell absorption edges of carbon (284 eV, λ = 4.4 nm) and oxygen (543 eV, λ = 2.3 nm) is directly obtained by the higher x-ray absorption by carbon rich structures compared to water [8]. Second, the density differences in biological samples results in different linear x-ray absorption coefficients providing information to identify cellular compartments. Lipid rich structures, for example, exhibit a higher absorption than water rich organelles [9]–[12]. Recent work using mouse adenocarcinoma cells demonstrated the high spatial resolution of 36 nm that can be achieved with the HZB-TXM soft x-ray microscope at the electron storage ring BESSY II and using the Bsoft software package for reconstructions of tomograms [7], [13].

The unicellular green alga *Chlamydomonas reinhardtii* is widely used as a model for the analysis of eukaryotic cell structure and function [14]. The first ultrastructural studies were carried out as early as in the 1950s [15]. *Chlamydomonas* contains a typical cup-shaped chloroplast that occupies about 40% of the cell volume. Starch deposits typically surround the pyrenoid, a Rubisco containing area [16], [17], and also accumulate between the thylakoid membranes [18]. Mitochondria appear as elongated organelles forming an interconnected network [19]. *Chlamydomonas reinhardtii* has two contractile vacuoles that fulfill structural tasks and are considered to be devices of osmoregulation especially under hypotonic conditions [20]. The nucleus is around 2–4 µm in diameter [21], [22], and Golgi stacks are mainly localized in the vicinity of the nucleus and reticulate ER [23]. Two flagella emerge from basal bodies at the anterior end. Basal bodies in green algae and their structural features resembling the centriole were extensively analyzed [24], [25]. Lipid bodies are often found in the cytosol and the plastid and play an import role in the storage of lipids [26]. While these studies revealed many structural details of single organelles, a high resolution picture of an entire *Chlamydomonas* cell is still lacking. Here, we used soft x-ray microscopy to generate whole cell tomograms.

## Materials and Methods

### Strains and Culture Conditions

*Chlamydomonas reinhardtii* cw15^+^ Dangeard (SAG 83.81) [27] and wild type strain CC125 [28] were cultured in 250 ml flasks at 25°C with a light-dark cycle of 14∶10 h and a photon flux density of 20 µE · m^−2^ · s^−1^ in TAP culture medium [29]. Cells were harvested after 5–7 days of growth.

### Sample Preparation

10 ml 5–7 day old suspension cultures were centrifuged at 300 g for 5 min, the pellet was resuspended in 1 ml of culture medium and incubated for 1 h at 20°C. 0.6 µl of the cell suspension was applied on nitrocellulose coated grids (type IFR-1, Gilder Grids, Grantham, UK). 100 nm fiducial gold markers were distributed on the support film of the grids before the samples were applied. Samples were then plunge frozen in liquid ethane and transferred into liquid nitrogen.

### Soft X-ray Microscopy

For the acquisition of tilt-series the HZB transmission x-ray microscope (TXM) at the U41-FSGM beamline at the electron storage ring BESSY II (Berlin, Germany) was used [30]. Tilt-series were recorded from −60° to +60° with 1° increment using x-rays with 2.43 nm wavelength (photon energy 510 eV). Samples were transferred under liquid nitrogen conditions into a Gatan (Pleasanton, CA, USA) 630 sample-holder and inserted into a modified FEI (Eindhoven, Netherlands) CompuStage. The condenser is an ellipsoidal glass capillary X-ray mirror from XRADIA Inc. (Pleasanton, CA, USA) [31] using a focal spot size of around 1 µm [32]. A high resolution zone plate objective with 901 zones and outermost zone width of dr_N_ = 25 nm providing a pixel size of 9.6 nm was used. This setup allows visualization of structures down to 17 nm in size [33]. To compensate for the increasing absorption at higher tilt angles the exposure time varied from 2 to 8 s from lower to higher tilt angles. The photon flux impinging the sample was about 7 · 10^8^ photons/(µm^2^ s 100 mA 0.01% BW). The projections for every tilt were recorded using a Peltier cooled, back thinned and direct illuminated 1340×1300 pixel soft x-ray CCD camera (Roper Scientific PI-SX 1300, Trenton, NJ, USA).

### Tomographic Reconstruction

Preprocessing was done using an average gain reference calculated from ten flatfield images taken under the same conditions. Each image of the tilt-series was divided by this average flatfield image for homogenization of the background and normalization to the beam current. Images from the preprocessed tilt-series were processed using the package Bsoft [34] and by using 100 nm fiducial gold particles for alignment. The second reconstruction approach was based on the fiducial less GPU powered alignment software Alignator [35]. As a third reconstruction approach IMOD was used [36].

### Segmentation and Data Analysis

The 3D x-ray tomograms were hand-segmented using the IMOD software package [36]. Video projections were calculated in IMOD and converted into.avi files using ImageJ [37]. Area and volume measurements and line plot profiles were also carried out with ImageJ.

## Results

### Sample Preparation and Data Acquisition

Two different algae strains were imaged using the HZB TXM setup: a *Chlamydomonas reinhardtii* wild type strain, CC-125 [28], and a cell wall-less mutant, cw15^+^ [27]. The cell wall-less mutant was chosen to optimize the imaging of cytoplasmic structures as the cell wall was expected to influence the x-ray absorbance. Samples were prepared by applying *Chlamydomonas* cell suspensions to grids seeded with 100 nm colloidal gold fiducial markers. The grid was then blotted and quickly frozen in liquid ethane. We recorded tilt series from −60° to +60° with 1° increment using x-rays with 2.43 nm wavelength. Depending on the defocus of the sample during rotation, we carried out a refocus of the sample after every 10 to 20 degrees of tilt. Tilt series were recorded for six wild type and eight cw15^+^ cells.

Several parameters turned out to be crucial to obtain high quality tomograms. First, the cells had to be applied to the grids at a concentration that avoids extensive overlap of the cells. When cells are closely packed (Fig. S1A) they will obstruct each other during tilting, and reconstructing the tilt-series results in a low quality tomogram. Another potential problem is that fiducials and the cell are not in focus at the same time (Fig. S1B). As the number of ice cracks sometimes increase during acquisition of the tilt series structural damage induced by these ice cracks may destroy the cell during image acquisition (Fig. S1C). About 5–10% of the cells were found to have damage caused by ice cracks. Furthermore, not all cells are preserved with the same quality during plunge freezing, and artefacts such as spider web-like structures may be visible within the cytoplasm (Fig. S1D), or plasmolysed cells may be observed (Fig. S1E). About 20% of the cells were affected by freezing damages. When cells are covered only by a very thin ice layer fiducials, grid bars, and the algal cells are in the same plane. However, these cells often show x-ray damage close to the ice surface (Fig. S1F). Thus, cell density, the absence of cellular damages and ice cracks, and the presence of an ice layer sufficiently thick to cover the cells are crucial factors for high quality tomograms.

**Figure 1:**
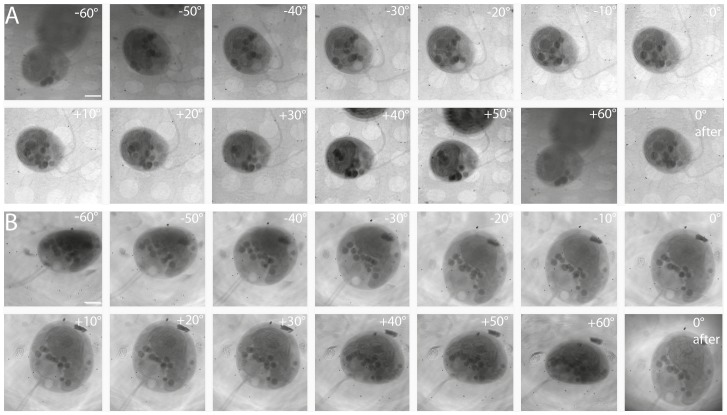
Soft x-ray dataset of *Chlamydomonas reinhardtii* wild type and cw15^+^ strain used for tomographic reconstruction. (A) Wild type strain tilt-series taken from −60° to +60° and 0° image after acquisition. Low tilt angles were exposed for 2 s, higher tilt (40° onwards) for 8 s. All angles provide good structural resolution and were all used for the reconstruction of the tomograms. Bar, 2 µm. (B) cw15^+^ strain tilt-series −60° to +60° and 0° image after acquisition were taken as above. Bar, 2 µm.

To prove that no x-ray damage in the cells occurred during tomographic data set acquisition we acquired 0° images before and after acquisition of the tilt-series (Fig. S1G-I). Compared to the wild type, cw15^+^ cells appeared flattened in the direction along the optical axis with a larger diameter in the x/y axes, probably because they adhere to the nitrocellulose film on the grid. Figure 1 shows the images acquired in representative tilt series for a wild type and a cw15^+^ cell at different tilt angles and the 0° tilt after acquisition. A sufficient number of gold particles are visible in all rotation angles within the field of view to permit an accurate image alignment (Fig. 1A, B). The cells appear well preserved and no freezing damage is noticed. Due to the high natural contrast provided by x-ray microscopy, cellular structures are visible in all angles of the tilt-series. These cells were chosen for further analysis.

### Alignment and Reconstruction Routines

**Figure 2:**
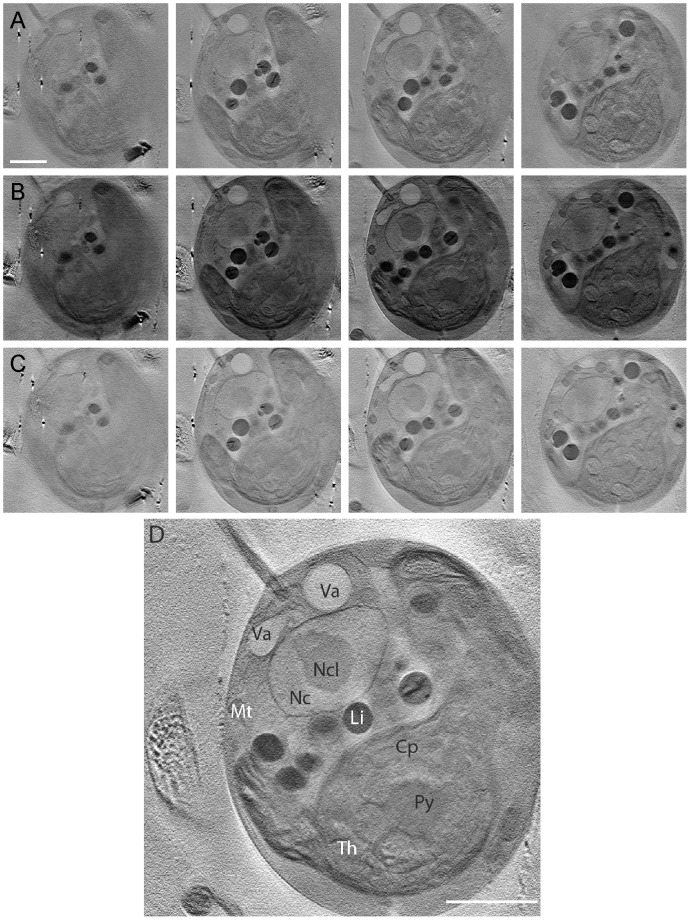
Tomographic reconstruction using IMOD, Bsoft and Alignator reconstruction software applied to the *Chlamydomonas* cw15^+^ dataset shown in Fig. 1B. (A) Representative tomographic sections using the fiducial based Etomo package included in IMOD. Bar, 2 µm. (B) Representative tomographic sections using the back-projection based alignment procedure Bsoft. (C) Representative tomographic sections using the fiducial less alignment approach Alignator. (D) Z-section generated with Alignator. A number of organelles can be identified: Chloroplast (Cp) with the starch containing pyrenoid (Py) and thylakoids (Th); nucleus (Nc) with nucleolus (Ncl); vacuoles (Va); mitochondria (Mt); lipid bodies (Li). Bar, 2 µm.

We used three different alignment approaches for 3D reconstruction: IMOD is a commonly used set of image processing programs for tomographic reconstruction and for 3D reconstruction of EM serial sections and optical sections [36]. Its etomo package uses fiducials for alignment, and data sets are calculated from a series of tilted views using the weighted back projection algorithm [38]. We used IMOD to reconstruct five different tilt series. Bsoft, another fiducial-based method, was programmed to analyze cryo tomography datasets which are characterized by low contrast and high noise [39]. Bsoft uses backprojection and a reciprocal space algorithm for the reconstruction. Four different tilt series were reconstructed with Bsoft. Alignator is a fiducial-less alignment approach that uses patches recognition [35]. It locates local areas in different micrographs that appear to correspond with different views of the object, and thus obviates the need of fiducials for 3D reconstruction. We selected two tomograms for reconstruction with all three softwares, including Alignator. Figure 2 shows four projections of a *Chlamydomonas* cw15^+^ tomogram reconstructed with IMOD (Fig. 2A, Video S1), Bsoft (Fig. 2B, Video S2) and Alignator (Fig. 2C, Video S3). The tomograms are similar in quality and resolve the whole cell in all three dimensions.

Alignments by IMOD depend on pixel size, the size of gold fiducials, sample thickness, and the precision of fiducial tracking. Alignment errors arise when the fiducial positions are not adequately described by the alignment model. IMOD delivers good results in thin ice layers, but has problems with the automatic recognition of fiducials at higher tilt angles and in thicker ice layers. Typical signs of these systematic errors are an elliptical appearance of the gold particles and halos in different directions. Furthermore, we found that tomograms with a limited number of fiducials, or fiducials which could not be followed over 120 tilt angles, deliver low-resolution tomograms with a mean residual error of over 0.7 pixels. IMOD delivers decent results with a residual error of 0.4 to 0.6 pixels in the fine alignment if at least four fiducials are found in all projections (Fig. 2A). Under optimal conditions, reconstruction of soft x-ray datasets using IMOD showed round gold particles with tails vertical to the rotation axis, a visual indicator of good alignment quality. The quality of the aligned series could be further increased by excluding some of the projections from the alignment. If no fiducials are present the alignment procedure has to be carried out using Midas, a manual alignment method included in IMOD, yielding results of varying quality (data not shown).

We found that Bsoft requires only a small number of gold particles to reconstruct complete volumes. The marker tracking with Bsoft for soft x-ray datasets was fully automatic and – in contrast to IMOD – required only minor manual changes. Furthermore, Bsoft does not need a manual refinement of the positions, which is required with IMOD particularly at high tilt angles. Especially for a low number of fiducials Bsoft allows the reconstruction with low residual errors. It causes fewer artefacts compared to programs that use the weighted backprojection algorithm, and produces a stronger contrast in the tomogram (Fig. 2B).

For alignment with Alignator 10,000 anchor points were randomly distributed over the tilt-series, the threshold for the hysteresis coefficient was set to 4 pixels, the length of the patch was set to 180 pixels, and the search length to 240 pixels. For all samples reconstructed with Alignator, a stability coefficient of 50 pixels was used. The hysteresis check guarantees the reproducibility of marker chase. Practically this means that the tracing of the markers does not depend on the direction of the search, i.e. from the positive or the negative tilt-direction. In general, and in normal cases the hysteresis check is a criterion which is being easily fulfilled. The hysteresis check has proved to be a powerful tool to reliably recognize pathological cases as for example extensive radiation damage, or a non-geometric movement of the sample. After alignment the quality of the trails was analyzed and linear trails were chosen for reconstruction. We found that Alignator produced high quality reconstructions, even in the absence of fiducials (Fig. 2C). Several cellular structures and organelles could be readily identified (Fig. 2D).

### 3D Reconstruction of Cellular Structures

We used tomograms reconstructed with Alignator for the analysis of the cellular architecture of *Chlamydomonas reinhardtii* (Videos S3, S4). The cellular organization and content of wild type and cell wall-less cw15^+^ cells appeared very similar. However, the mutant was flattened due to its adhesion to the grid surface (4 µm in the z axis for the cw15^+^ cell versus 7 µm for the wild type cell). The cup-shaped chloroplast was the most prominent cell organelle in both cells. Several of its internal features could be recognized, including thylakoids and the pyrenoid with its starch deposits. Other clearly discernible structures are the nucleus, the nucleolus, and vacuoles. Several spherical structures were seen in the cytoplasm. Because of their high x-ray absorption we consider it likely that they represent lipid bodies. Mitochondria were clearly visible. However, their profiles could not be traced in all planes of the tomogram. Golgi stacks and ER were more difficult to visualize.

**Figure 3:**
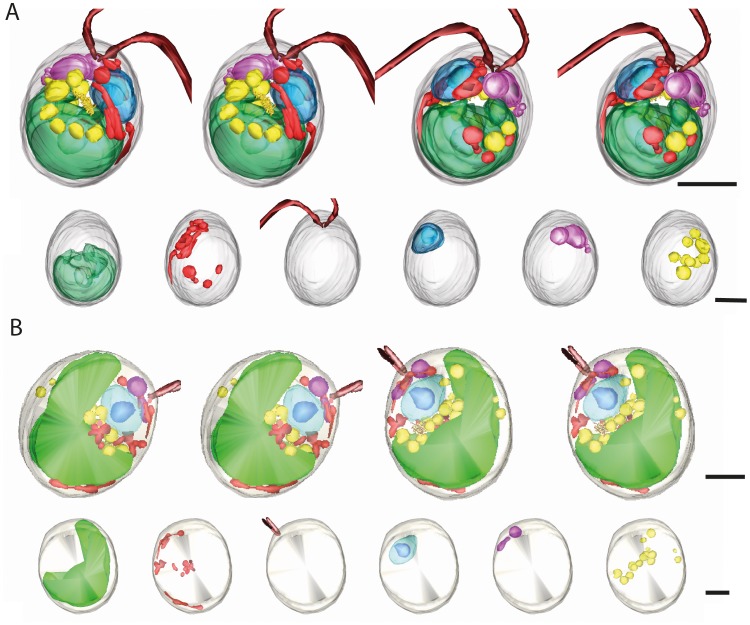
Segmentation of cellular structures in *Chlamydomonas* cells. (A) Segmentation of a wild type cell tomogram. Top, stereo pairs in front view and after 180° rotation. Bottom, from left to right: chloroplast with pyrenoid; mitochondria; flagella; nucleus with nucleolus; vacuoles; lipid bodies. Bars, 2 µm. The tomogram and segmented volumes of the same cell are shown in Videos S4 and S5 (B) Segmentation of a cw15^+^ cell tomogram. Segmented volumes are presented as in A. The tomogram and segmented volumes of the same cell are shown in Videos S3 and S6.

We segmented selected organelles and cell structures in the wild type tomogram to obtain quantitative information about the organelle distribution in whole cells. Fig. 3A and Video S5 show reconstructed and segmented volumes of the wild type cell, and a series of segmented cell organelles. We measured a total cell volume of 122 µm^3^. The chloroplast fills about 30% of the cell volume, and its starch-containing pyrenoid could be reconstructed. The nucleus fills 5% of the cell volume, and the nucleolus could be clearly seen. The cell contains two large and several small vacuoles that are located close to the basal apparatus and together make up 2.7% of the cell volume. Lipid bodies are dispersed in the cytoplasm and fill 2.8% of the cell volume. Mitochondria are located in the cell periphery. However, it was not possible to determine their volume as it was difficult to track the mitochondrial profiles in all planes of the tomogram. Finally, the cell's two flagella together with the basal body and the distal connecting fiber could be reconstructed and segmented. The cell wall-less cw15^+^ cell was slightly larger (135 µm^3^). Segmentation of cellular structures revealed a very similar organelle content (Fig. 3B, Video S6). The observed organelle volumes of the wild type and cw15^+^ cell correspond well to data in the literature [40].

### Ultrastructural Details

**Figure 4:**
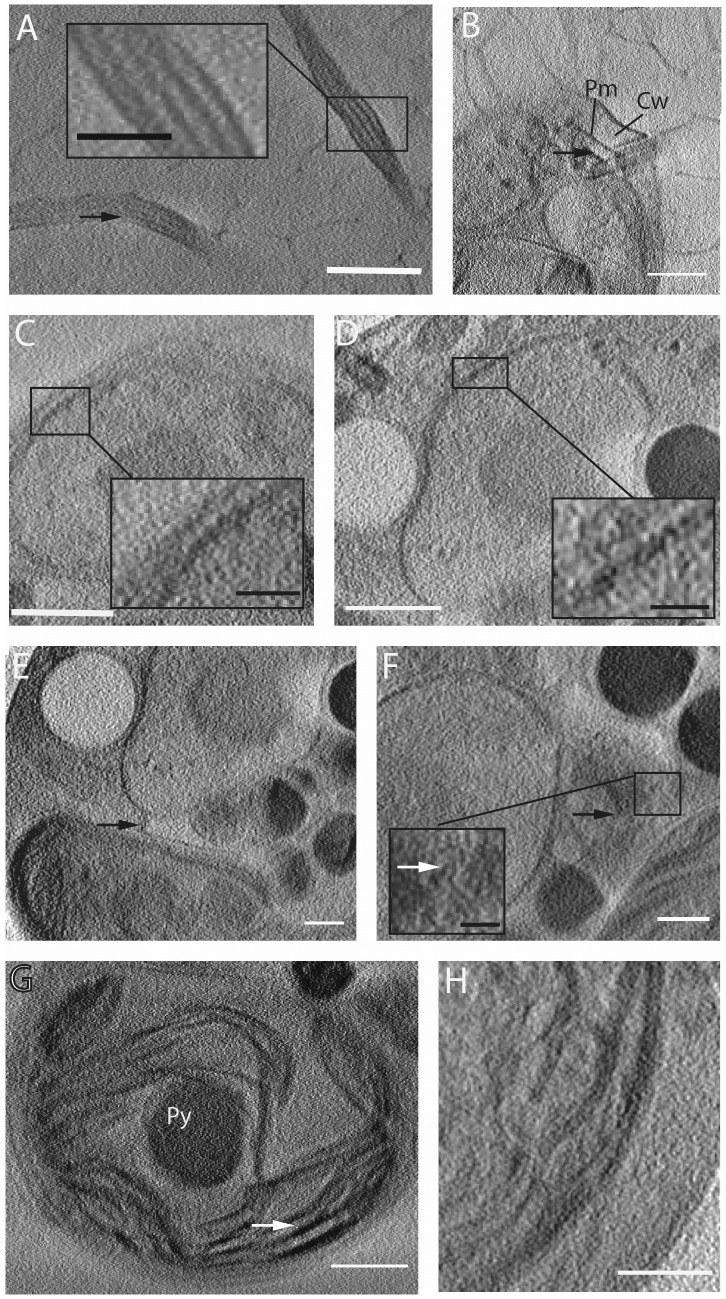
Ultrastructural details in wild type and cw15^+^ cell tomograms. (A) Sections of flagella in the tomogram of the wild type cell. The arrow indicates a microtubule doublet. Bar, 1 µm. Inset: enlarged view showing flagellar membrane and microtubule doublets; bar, 200 nm. (B) Basal apparatus with distal connecting fiber (arrow) connecting the two basal bodies in the wild type cell. Pm, plasma membrane; Cw, cell wall. Bar, 1 µm. (C) Nuclear envelope membranes in the tomogram of the wild type cell. Bar, 1 µm. Inset: enlarged view showing inner and outer nuclear envelope membrane; bar, 200 nm. (D) Nuclear envelope membranes in the tomogram of the cw15^+^ cell. Bar, 1 µm. Inset: enlarged view showing inner and outer nuclear envelope membrane; bar, 200 nm. (E) Putative ER membrane (arrow) emanating from the nuclear envelope of the cw15^+^ cell. Bar, 500 nm. (F) Contrast rich cisternae structure (black arrow) which could be part of the Golgi apparatus in the cw15^+^ cell. Bar, 500 nm. Inset, possible vesicle budding site (white arrow). Bar, 200 nm. (G) Ultrastructural details of the chloroplast of the cw15^+^ cell: thylakoids and granae (arrow) and pyrenoid (Py). Bar, 1 µm. (H) Thylakoids and plastid envelope membranes of the chloroplast of the cw15^+^ cell. Bar, 500 nm.

Soft x-ray tomography revealed several ultrastructural details in *Chlamydomonas* cells. Axonemal microtubule doublets could be visualized in the flagella (Fig. 4A). To determine the spatial resolution we generated surface plots of grey values and measured the sizes and distances of the cellular structures that could be resolved within the flagella (Fig. S2). The flagellar diameter varied from 219 to 329 nm. These variations are likely due to flattening effects of flagella during cryofixation [41]. The distance of the microtubule doublets ranged from 28 to 35 nm, and the diameter of microtubule doublets measured to be 25 to 41 nm. Thus, we were able to resolve cellular structures at a resolution of less than 30 nm under optimal conditions. These values fit well to the dimensions of flagellar structures observed by electron microscopy [25].

The distal connecting fiber of the basal apparatus could be clearly resolved (Fig. 4B). Structural details visualized in the nucleus are the nucleolus and the nuclear envelope. The two membranes of the nuclear envelope could be separated in some sections of the tomograms, and the observed thickness of the nuclear envelope was about 45–50 nm. This corresponds to the smallest distance that could be resolved in the interior of the cell (Fig. 4C, D). ER membranes are very difficult to visualize – in *Chlamydomonas* the ER is limited to a small extension directly adjacent to the nucleus, which could be identified only in a few z-slices (Fig. 4E). The Golgi apparatus was vaguely visible close to the nucleus. The cisternal shape (Fig. 4F, black arrow) and possibly a budding vesicle could be seen (Fig. 4F, white arrow). However, the size of these structures is close to the limit of resolution (the width of one cisterna corresponds to 2–4 pixels) preventing segmentation and 3D reconstruction. Structural details that could be readily identified in the chloroplast are thylakoids and the pyrenoid (Fig. 4G). Single thylakoids could be resolved using x-ray microscopy (Fig. 4H).

## Discussion

In the literature, several organisms, such as bacteria, viruses, yeast and mammalian cells, were analyzed by x-ray tomography in recent years illustrating the potential of x-ray microscopy [9]–[11], [42]–[46]. Here we demonstrate that soft x-ray imaging of cryo-preserved samples produces high quality, high resolution tomographic data from unstained unicellular algae. The maximum resolution was estimated to be less than 30 nm in the flagella and below 50 nm in the cell body, as measured by a point-to-point distance. These values obtained for *Chlamydomonas* compare well with the resolution of 36 nm reported for mammalian tissue culture cells [7]. Nuclear envelope membranes could be resolved in both the wild type and the cw15^+^ cell suggesting that the presence of the cell wall does not have a major effect on the resolution of the tomogram.

The resolution was estimated as proposed by Cardone et al. [47] using Fourier shell correlation on two reconstructions obtained from odd and even projections and by the point-to-point distance of known objects. The first method yielded a resolution of ∼210 nm, while it is apparent that much smaller structures can be observed in the tomograms. This discrepancy can be explained by the fact that we examined a relatively thick specimen and the recorded 120 projections are not fulfilling the Crowther criterion at a resolution better than ∼210 nm [48]. However, this is a prerequisite for an accurate estimation of the resolution by this method. The point-to-point distance of the smallest resolved objects yields a resolution of ∼30 nm which is a reliable and conservative method to determine the resolution in whole cell x-ray tomograms.

The most important procedure for the reconstruction of tomograms providing detailed information within the resolution limit is based on the alignment step. Alignment of x-ray tomography data sets differs in some aspects from the alignment of electron tomography data sets. The first factor is the presence of fiducial markers within the tilt-series. For reconstruction procedures using the software packages like IMOD [36] and Bsoft [34] the presence of a sufficient number of fiducials at all tilt angles is crucial for obtaining tomograms with low error values below 0.7 pixels. The thickness of the ice layer as well as contrast rich structures within the area of interest makes it sometimes difficult to locate the exact position of all fiducials. In particular for IMOD in high tilt angles the selected fiducials have to be marked manually. Thin ice layers have the advantage of fiducials and cells being in the same focal plane, but sometimes the specimens are not totally embedded in the ice which tends to produce structural damages during data acquisition. Fiducial markers in the upper ice layer sometimes change their position during acquisition due to damage of this upper ice layer and increase the reconstruction error dramatically. The shift of gold beads or the low number of fiducials and the resulting poor quality of the reconstructed volumes makes it necessary to consider non-fiducial based alignment methods. We show that Alignator [35] is ideally suited to produce high quality reconstructions for soft x-ray tilt-series. It obviates the need to add fiducials, which sometimes turns out to be difficult. Moreover, fiducials are often hardly visible at high tilt angles. Thus, Alignator provides an alternative to the commonly used fiducial based alignment methods when samples show low numbers of fiducials and the identification of these markers causes problems at high tilt angles.

*Chlamydomonas reinhardtii* is a widely used model organism in cell and molecular biology. One of our aims was to demonstrate that soft x-ray tomography of *Chlamydomonas* cells provides high spatial resolution and closes the gap between light and electron microscopy. By combining soft x-ray imaging and using different alignment approaches we were able to image a number of cellular structures and compartments using only the natural contrast of the sample. We showed ultrastructural features of the basal apparatus including the distal connecting fiber between the two basal bodies. This structure could be imaged so far only by electron microscopy using sectioning methods [25]. Furthermore, we were able to resolve ultrastructural details such as thylakoids, the two membranes of the nuclear envelope, and microtubule doublets in the flagella. Moreover, the whole cell tomograms allowed the determination of volumes and copy number of several organelles, including plastid, nucleus, vacuoles, and lipid bodies. Nanoscale x-ray tomography could be of considerable interest for plant cell research in general, as it should be possible to image also higher plant cells, such as protoplasts or pollen tubes. Moreover, sections of various tissues up to a thickness of 15 µm can be applied to the grids [45], [49]. In the future, the development of new zone plates with the combined use of third order diffraction imaging will further improve the resolution of x-ray microscopy [6], [33], and a correlative light microscope setup currently mounted on the soft x-ray microscope permits co-localization studies using fluorescent markers [30].

## Supporting Information

Figure S1 **Tilt-series selection – Finding the right cell.** Bars 2 µm. (A) Densely packed *Chlamydomonas* cells; tilting would result in overlap of cells during tilt. (B) Cells embedded in thick ice; simultaneous focus of fiducials and the cell is not possible, resulting in a low quality tomogram. (C) Area showing a prominent ice crack within the field of view (arrow); such cracks often increase during x-ray imaging and lead to structural damage during acquisition. (D) Freezing damage visible as spider web-like structures occurred during plunge freezing (arrows). (E) Plasmolysed cell. (F) Cell embedded in thin ice – fiducials and the cell are clearly visible; however, thin ice often results in structural damage (arrow) during tilt-series collection if the cell is not completely embedded in ice. (G) 0° tilt image before and after acquisition of a tilt-series. During acquisition of the tilt-series an ice crack formed, and data acquisition was aborted. Ice cracks tend to expand over time in the x-ray beam. Especially the plastids and the cell wall show first signs of deformation. The black arrow shows the newly formed ice crack, the white arrow shows the resulting structural damages. (H) cw15^+^ cell used for the dataset shown in Fig. 1B: Images of 0° tilt before and after acquisition of tilt-series; no changes of cellular structure and structural damage within the cell are visible. (I) Wild type cell used for the dataset shown in Fig. 1A: 0° tilt before and after the acquisition of the tilt-series: despite of the thin ice no structural changes can be observed.
(TIF)

Figure S2 **Determination of resolution in flagella of the wild type** ***Chlamydomonas*** **cell.** Left, micrographs of x/y planes of similar areas in flagella reconstructed with IMOD, Bsoft, and Alignator are shown. IMOD and Alignator tomograms were reconstructed without binning, the Bsoft tomogram was reconstructed with two-fold binning. Bars, 200 nm. Right, line plot profiles of grey values generated with ImageJ. The lines used to generate the plots are indicated by a yellow line in the micrographs. The smallest structure resolved in the Alignator tomogram is indicated in red (22.5 nm).
(TIF)

Video S1 **Tomogram of the cw15^+^** ***Chlamydomonas reinhardtii*** **cell reconstructed with IMOD.**
(AVI)

Video S2 **Tomogram of the cw15^+^** ***Chlamydomonas reinhardtii*** **cell reconstructed with Bsoft.**
(AVI)

Video S3 **Tomogram of the cw15^+^** ***Chlamydomonas reinhardtii*** **cell reconstructed with Alignator.**
(AVI)

Video S4 **Tomogram of the wild type** ***Chlamydomonas reinhardtii*** **cell reconstructed with Alignator.**
(AVI)

Video S5 **360° rotation of the segmented wild type** ***Chlamydomonas reinhardtii*** **cell.**
(AVI)

Video S6 **360° rotation of the segmented cw15^+^** ***Chlamydomonas reinhardtii*** **cell.**
(AVI)

## References

[1] Hell SW (2007) Far-field optical nanoscopy. Science 316: 1153–1158. 17525330 10.1126/science.1137395

[2] Hurbain I, Sachse M (2011) The future is cold: cryo-preparation methods for transmission electron microscopy of cells. Biol Cell 103: 405–420. 21812762 10.1042/BC20110015

[3] Lacomble S, Vaughan S, Gadelha C, Morphew MK, Shaw MK, et al (2009) Three-dimensional cellular architecture of the flagellar pocket and associated cytoskeleton in trypanosomes revealed by electron microscope tomography. J Cell Sci 122: 1081–1090. 19299460 10.1242/jcs.045740 PMC2714436

[4] Frey TG, Perkins GA, Ellisman MH (2006) Electron tomography of membrane-bound cellular organelles. Annu Rev Biophys Biomol Struct 35: 199–224. 16689634 10.1146/annurev.biophys.35.040405.102039

[5] Henderson GP, Gan L, Jensen GJ (2007) 3-D ultrastructure of *O. tauri*: electron cryotomography of an entire eukaryotic cell. PLoS One 2: e749. 17710148 10.1371/journal.pone.0000749 PMC1939878

[6] Rehbein S, Guttmann P, Werner S, Schneider G (2012) Characterization of the resolving power and contrast transfer function of a transmission X-ray microscope with partially coherent illumination. Opt Express 20: 5830–5839. 22418460 10.1364/OE.20.005830

[7] Schneider G, Guttmann P, Heim S, Rehbein S, Mueller F, et al (2010) Three-dimensional cellular ultrastructure resolved by X-ray microscopy. Nat Methods 7: 985–987. 21076419 10.1038/nmeth.1533 PMC7337972

[8] Kirz J, Jacobsen C, Howells M (1995) Soft X-ray microscopes and their biological applications. Q Rev Biophys 28: 33–130. 7676009 10.1017/s0033583500003139

[9] McDermott G, Le Gros MA, Knoechel CG, Uchida M, Larabell CA (2009) Soft X-ray tomography and cryogenic light microscopy: the cool combination in cellular imaging. Trends Cell Biol 19: 587–595. 19818625 10.1016/j.tcb.2009.08.005 PMC3276488

[10] Uchida M, McDermott G, Wetzler M, Le Gros MA, Myllys M, et al (2009) Soft X-ray tomography of phenotypic switching and the cellular response to antifungal peptoids in *Candida albicans* . Proc Natl Acad Sci U S A 106: 19375–19380. 19880740 10.1073/pnas.0906145106 PMC2780763

[11] Uchida M, Sun Y, McDermott G, Knoechel C, Le Gros MA, et al. (2010) Quantitative analysis of yeast internal architecture using soft X-ray tomography. Yeast. 10.1002/yea.1834 PMC3404734 21360734

[12] Weiss D, Schneider G, Niemann B, Guttmann P, Rudolph D, et al (2000) Computed tomography of cryogenic biological specimens based on X-ray microscopic images. Ultramicroscopy 84: 185–197. 10945329 10.1016/s0304-3991(00)00034-6

[13] Muller WG, Bernard Heymann J, Nagashima K, Guttmann P, Werner S, et al (2012) Towards an atlas of mammalian cell ultrastructure by cryo soft X-ray tomography. J Struct Biol 177: 179–192. 22155291 10.1016/j.jsb.2011.11.025 PMC3288423

[14] Harris EH (2001) Chlamydomonas as a model organism. Annu Rev Plant Physiol Plant Mol Biol 52: 363–406. 11337403 10.1146/annurev.arplant.52.1.363

[15] Sager R, Palade GE (1954) Chloroplast structure in green and yellow strains of *Chlamydomonas* . Exp Cell Res 7: 584–588. 13220605 10.1016/s0014-4827(54)80107-8

[16] Nozaki H, Onishi K, Morita E (2002) Differences in pyrenoid morphology are correlated with differences in the rbcL genes of members of the *Chloromonas* lineage (volvocales, chlorophyceae). J Mol Evol 55: 414–430. 12355262 10.1007/s00239-002-2338-9

[17] Morita E, Abe T, Tsuzuki M, Fujiwara S, Sato N, et al (1998) Presence of the CO2-concentrating mechanism in some species of the pyrenoid-less free-living algal genus *Chloromonas* (Volvocales, Chlorophyta). Planta 204: 269–276. 9530871 10.1007/s004250050256

[18] Hummel E, Osterrieder A, Robinson DG, Hawes C (2010) Inhibition of Golgi function causes plastid starch accumulation. J Exp Bot 61: 2603–2614. 20423939 10.1093/jxb/erq091 PMC2882258

[19] Osafune T, Mihara S, Hase E, Ohkuro I (1975) Electron microscope studies of the vegetative cellular life cycle of *Chlamydomonas reinhardtii* dangeard in synchronous culture. III. Three-dimensional structures of mitochondria in the cells at intermediate stages of the growth phase of the cell cycle. J Electron Microsc (Tokyo) 24: 247–252. 1223238

[20] Ettl H (1976) About the progress of chloroplast division in *Chlamydomonas* Protoplasma. 88: 75–84. 10.1007/BF01280361 1273307

[21] Colon-Ramos DA, Salisbury JL, Sanders MA, Shenoy SM, Singer RH, et al (2003) Asymmetric distribution of nuclear pore complexes and the cytoplasmic localization of beta2-tubulin mRNA in *Chlamydomonas reinhardtii* . Dev Cell 4: 941–952. 12791277 10.1016/s1534-5807(03)00163-1

[22] Sager R (1955) Inheritance in the Green Alga *Chlamydomonas reinhardtii* . Genetics 40: 476–489. 17247567 10.1093/genetics/40.4.476 PMC1209736

[23] Hummel E, Schmickl R, Hinz G, Hillmer S, Robinson DG (2007) Brefeldin A action and recovery in *Chlamydomonas* are rapid and involve fusion and fission of Golgi cisternae. Plant Biol (Stuttg) 9: 489–501. 17301935 10.1055/s-2006-924759

[24] Geimer S, Teltenkotter A, Plessmann U, Weber K, Lechtreck KF (1997) Purification and characterization of basal apparatuses from a flagellate green alga. Cell Motil Cytoskeleton 37: 72–85. 9142440 10.1002/(SICI)1097-0169(1997)37:1<72::AID-CM7>3.0.CO;2-J

[25] O'Toole ET, Giddings TH, McIntosh JR, Dutcher SK (2003) Three-dimensional organization of basal bodies from wild-type and delta-tubulin deletion strains of Chlamydomonas reinhardtii. Mol Biol Cell 14: 2999–3012. 12857881 10.1091/mbc.E02-11-0755 PMC165693

[26] Fan J, Andre C, Xu C (2011) A chloroplast pathway for the de novo biosynthesis of triacylglycerol in *Chlamydomonas reinhardtii* . FEBS Lett 585: 1985–1991. 21575636 10.1016/j.febslet.2011.05.018

[27] Davies DR (1972) Cell wall organisation in Chlamydomonas reinhardi. The role of extra-nuclear systems. Mol Gen Genet 115: 334–348. 5033051 10.1007/BF00333172

[28] Pröschold T, Harris EH, Coleman AW (2005) Portrait of a species: Chlamydomonas reinhardtii. Genetics 170: 1601–1610. 15956662 10.1534/genetics.105.044503 PMC1449772

[29] Gorman DS, Levine RP (1966) Photosynthetic Electron Transport Chain of Chlamydomonas reinhardi. V. Purification and Properties of Cytochrome 553 and Ferredoxin. Plant Physiol 41: 1643–1647. 16656452 10.1104/pp.41.10.1643 PMC550587

[30] Schneider G, Guttmann P, Rehbein S, Werner S, Follath R (2012) Cryo X-ray microscope with flat sample geometry for correlative fluorescence and nanoscale tomographic imaging. J Struct Biol 177: 212–223. 22273540 10.1016/j.jsb.2011.12.023

[31] Zeng X, Duewer F, Feser M, Huang C, Lyon A, et al (2008) Ellipsoidal and parabolic glass capillaries as condensers for x-ray microscopes. Appl Opt 47: 2376–2381. 18449303 10.1364/ao.47.002376

[32] Guttmann P, Zheng X, Feser M, Yun W, Schneider G (2009) Ellipsidoidal capillary capillary as condenser for the BESSY full-field X-ray microscope. Journal of Physics Conference series: 012064.

[33] Rehbein S, Heim S, Guttmann P, Werner S, Schneider G (2009) Ultrahigh-resolution soft-x-ray microscopy with zone plates in high orders of diffraction. Phys Rev Lett 103: 110801. 19792359 10.1103/PhysRevLett.103.110801

[34] Heymann JB, Cardone G, Winkler DC, Steven AC (2008) Computational resources for cryo-electron tomography in Bsoft. J Struct Biol 161: 232–242. 17869539 10.1016/j.jsb.2007.08.002 PMC2409064

[35] Castano-Diez D, Scheffer M, Al-Amoudi A, Frangakis AS (2010) Alignator: a GPU powered software package for robust fiducial-less alignment of cryo tilt-series. J Struct Biol 170: 117–126. 20117216 10.1016/j.jsb.2010.01.014

[36] Kremer JR, Mastronarde DN, McIntosh JR (1996) Computer visualization of three-dimensional image data using IMOD. J Struct Biol 116: 71–76. 8742726 10.1006/jsbi.1996.0013

[37] Abramoff MD, Magalhaes PJ, Ram SJ (2004) Image Processing with ImageJ. Biophotics international 11: 36–42.

[38] Gilbert PF (1972) The reconstruction of a three-dimensional structure from projections and its application to electron microscopy. II. Direct methods. Proc R Soc Lond B Biol Sci 182: 89–102. 4403086 10.1098/rspb.1972.0068

[39] Heymann JB, Belnap DM (2007) Bsoft: image processing and molecular modeling for electron microscopy. J Struct Biol 157: 3–18. 17011211 10.1016/j.jsb.2006.06.006

[40] Harris EH (2009) The Chlamydomonas Source Book - Volume 1. Oxford, Burlington, San Diego. 444 p.

[41] Bui KH, Pigino G, Ishikawa T (2011) Three-dimensional structural analysis of eukaryotic flagella/cilia by electron cryo-tomography. J Synchrotron Radiat 18: 2–5. 21169680 10.1107/S0909049510036812 PMC3004243

[42] Larabell CA, Le Gros MA (2004) X-ray tomography generates 3-D reconstructions of the yeast, *Saccharomyces cerevisiae*, at 60-nm resolution. Mol Biol Cell 15: 957–962. 14699066 10.1091/mbc.E03-07-0522 PMC363052

[43] Uchida M, Sun Y, McDermott G, Knoechel C, Le Gros MA, et al (2011) Quantitative analysis of yeast internal architecture using soft X-ray tomography. Yeast 28: 227–236. 21360734 10.1002/yea.1834 PMC3404734

[44] Carrascosa JL, Chichon FJ, Pereiro E, Rodriguez MJ, Fernandez JJ, et al (2009) Cryo-X-ray tomography of vaccinia virus membranes and inner compartments. J Struct Biol 168: 234–239. 19616103 10.1016/j.jsb.2009.07.009

[45] Schneider G (2003) X-ray microscopy: methods and perspectives. Anal Bioanal Chem 376: 558–561. 12811455 10.1007/s00216-003-2007-x

[46] Chichon FJ, Rodriguez MJ, Pereiro E, Chiappi M, Perdiguero B, et al (2012) Cryo X-ray nano-tomography of vaccinia virus infected cells. J Struct Biol 177: 202–211. 22178221 10.1016/j.jsb.2011.12.001 PMC7119024

[47] Cardone G, Grünewald K, Steven AC (2005) A resolution criterion for electron tomography based on cross-validation. J Struct Biol 151: 117–129. 15964766 10.1016/j.jsb.2005.04.006

[48] Crowther RA, DeRosier DJ, Klug A (1970) The reconstruction of a three-dimensional structure from projections and its application to electron microscopy. Proc. Roy. Soc. Lond. A 317: 319–340. 10.1098/rspb.1972.0068 4403086

[49] Schmahl G, Rudolph D, Niemann B, Guttmann P, Thieme J, et al (1996) X-ray microscopy. Naturwissenschaften 83: 61–70. 8668229

