# Supplementary figures and images for: 3D Ultrastructural Organization of Whole Chlamydomonas reinhardtii Cells Studied by Nanoscale Soft X-Ray Tomography
Source: PLoS One. 2012 Dec 31;7(12):e53293. doi: 10.1371/journal.pone.0053293 (PMC3534036; doi:10.1371/journal.pone.0053293)

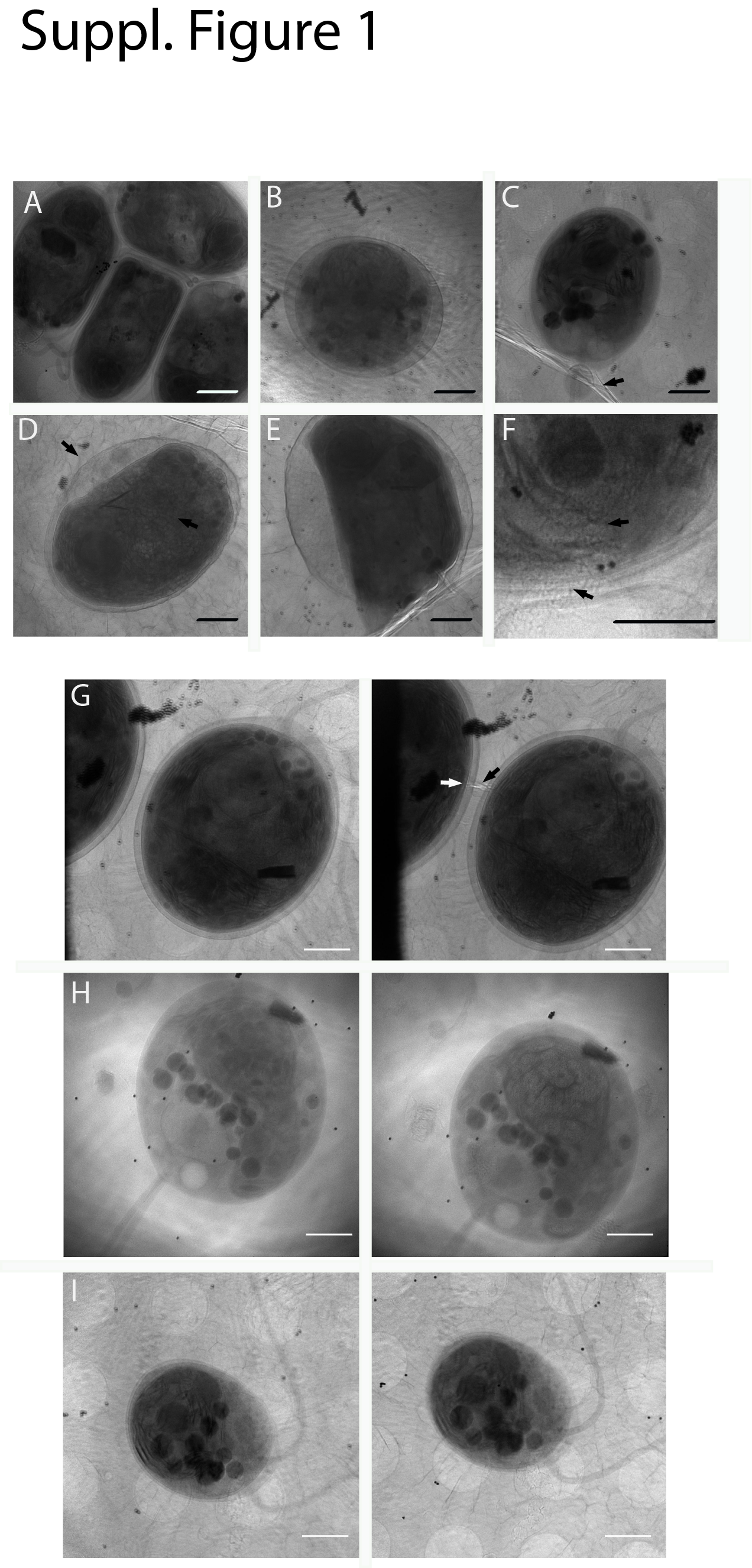

Supplement: Figure S1 — Tilt-series selection – Finding the right cell. Bars 2 µm. (A) Densely packed Chlamydomonas cells; tilting would result in overlap of cells during tilt. (B) Cells embedded in thick ice; simultaneous focus of fiducials and the cell is not possible, resulting in a low quality tomogram. (C) Area showing a prominent ice crack within the field of view (arrow); such cracks often increase during x-ray imaging and lead to structural damage during acquisition. (D) Freezing damage visible as spider web-like structures occurred during plunge freezing (arrows). (E) Plasmolysed cell. (F) Cell embedded in thin ice – fiducials and the cell are clearly visible; however, thin ice often results in structural damage (arrow) during tilt-series collection if the cell is not completely embedded in ice. (G) 0° tilt image before and after acquisition of a tilt-series. During acquisition of the tilt-series an ice crack formed, and data acquisition was aborted. Ice cracks tend to expand over time in the x-ray beam. Especially the plastids and the cell wall show first signs of deformation. The black arrow shows the newly formed ice crack, the white arrow shows the resulting structural damages. (H) cw15+ cell used for the dataset shown in Fig. 1B: Images of 0° tilt before and after acquisition of tilt-series; no changes of cellular structure and structural damage within the cell are visible. (I) Wild type cell used for the dataset shown in Fig. 1A: 0° tilt before and after the acquisition of the tilt-series: despite of the thin ice no structural changes can be observed. (TIF) [file pone.0053293.s001.tif]

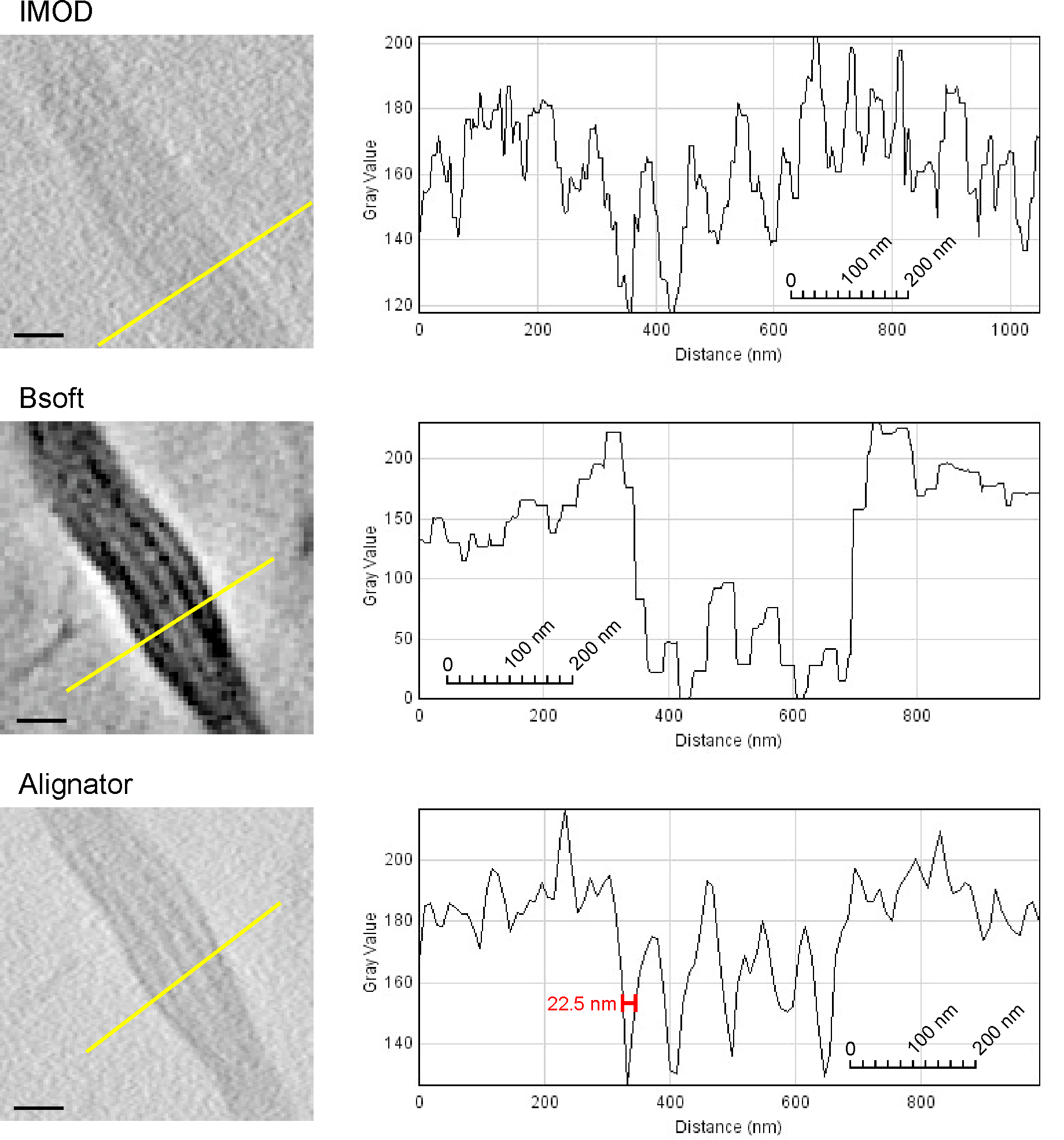

Supplement: Figure S2 — Determination of resolution in flagella of the wild type Chlamydomonas cell. Left, micrographs of x/y planes of similar areas in flagella reconstructed with IMOD, Bsoft, and Alignator are shown. IMOD and Alignator tomograms were reconstructed without binning, the Bsoft tomogram was reconstructed with two-fold binning. Bars, 200 nm. Right, line plot profiles of grey values generated with ImageJ. The lines used to generate the plots are indicated by a yellow line in the micrographs. The smallest structure resolved in the Alignator tomogram is indicated in red (22.5 nm). (TIF) [file pone.0053293.s002.tif]
